# Impact of family doctors on gradient utilization of health services among diabetic patients: evidence from a real-world study

**DOI:** 10.3389/frhs.2025.1618955

**Published:** 2025-08-22

**Authors:** Yin Fan, Mengyun Sui, Leiyu Shi, Long Xue, Su Xu

**Affiliations:** ^1^ Johns Hopkins University, Baltimore, MD, United States; ^2^ Shanghai Municipal Center for Disease Control & Prevention, Shanghai, China; ^3^ Bloomberg School of Public Health, Johns Hopkins University, Baltimore, MD, United States; ^4^ Huashan Hospital Fudan University, Shanghai, China; ^5^ Shangha Municipal Health Commission, Shanghai, China

**Keywords:** family doctor system, contracted patients, diabetic patients, outpatient visits, inpatient

## Abstract

**Objective:**

The family doctor system plays a crucial role in promoting the gradient utilization of health resources. However, empirical evidence regarding the use of health services across different levels of care by diabetic patients under family doctor contracts remains limited. This study aimed to investigate the impact of the family doctor system on the gradient utilization of health services among diabetic patients using real-world data.

**Methods:**

We conducted an eight-year cohort study in Shanghai from 2014 to 2021, with a final sample size of 491,674 participants, including 459,600 contracted and 32,074 non-contracted patients. We employed inverse probability weighted regression adjustment (IPWRA) and zero-inflated negative binomial regression models to estimate the net effects. Among contracted patients, 52.08% were female, with an average age of 66.31 years; in comparison, non-contracted patients were younger, and over 60% resided in urban areas. The annual number of outpatient and inpatient visits was 32.47 and 0.42 for contracted patients, and 34.63 and 0.35 for non-contracted patients, respectively.

**Results:**

Study results showed that, outpatient visits decreased across all levels of hospital (coef. = −7.37%, IRR = 0.92 *P* < 0.01), with a more pronounced reduction in secondary and tertiary hospitals compared to community health centers. This translated to a notable decrease of 2.43 days in the total number of outpatient visits. Conversely, hospitalization rates increased, particularly in community health centers (coef. = 26.88%, IRR = 1.30, *P* < 0.01). Overall, the data suggest that having a family doctor is associated with reduced outpatient visits, especially in higher-level hospitals, while hospitalizations are more concentrated in community health centers.

**Conclusion:**

Hospitalization rates can be reduced through targeted measures: strengthening early screening for diabetic complications; implementing a health-focused digital management system with outcomes-linked performance evaluations; enhancing clinical decision support and re mote monitoring systems to enable timely interventions by family doctors; developing clear referral protocols to minimize unnecessary hospital admissions; and conducting regular competency training for primary care providers.

## Introduction

According to the International Diabetes Federation (IDF) Diabetes Atlas, 10th Edition (2021), approximately 537 million adults aged 20–79 years are living with diabetes globally, accounting for nearly 10% of the world's population. China bears the highest burden, with an estimated 140 million adults affected in 2021, projected to rise to 170 million by 2045 (1). Without effective management, diabetes can lead to acute and chronic complications, resulting in increased hospitalizations and healthcare costs (2, 3), thereby posing a significant challenge to healthcare systems.

In most countries, primary care plays a central role in diabetes management. Family doctors act as “gatekeepers,” establishing contractual relationships with patients and providing standardized services including risk assessment, disease screening, chronic disease management, follow-up interventions, and health education (4). The family doctor contracting system facilitates primary care engagement and two-way referrals, promoting the rational and hierarchical utilization of healthcare resources and reducing resource wastage (5).

It is generally hypothesized that diabetic patients receiving family doctor services are more likely to seek care at primary healthcare institutions, thereby reducing visits to secondary and tertiary hospitals and fostering a graded healthcare utilization pattern. Moreover, family doctor services are regarded as key to improving health outcomes and reducing healthcare costs. However, this premise remains debated among both patients and physicians. A considerable body of research from various countries has explored the impact of the family doctor system on outpatient visits, emergency department visits, hospitalizations, and avoidable admissions (6, 7). For instance, in Australia, potentially preventable hospitalizations were significantly lower among patients with general practitioner (GP) utilization compared to those without (IRR = 0.67) (8), whereas Welberry et al. found no evidence that GP management significantly reduced avoidable hospitalizations (9). In the United States, In America, the pilot practices found that GP collaborative practice has statistically significantly lower rates of all-cause hospitalization, emergency department visits and higher rates of primary care visits, little evidence of substantial changes in the use of specialty services by adults in the first 18 months after the elimination of gatekeeping (10, 11). In China, studies have shown that family doctor contracting increased follow-up and outpatient visits among patients with hypertension but not diabetes, while in Taiwan province, the Family Physician Integrated Care Program reduced hospital admissions in the long term (12). In Europe, research by Van Loenen et al. found that countries with better perceived access to care paradoxically had higher rates of hospital admissions for long-term diabetes complications (13).

In 2011, S City launched the pilot family doctor contracting service in 10 administrative districts, expanding citywide by 2013. In 2015, S City introduced the innovative “1 + 1 + 1” combined contracting model, allowing residents to voluntarily select a community health center, a district-level hospital, and a municipal hospital (including traditional Chinese medicine hospitals) for integrated care. This pioneering approach empowers residents with the flexibility to voluntarily choose a community, district, and central hospital for combined contracting (14–16).

Three main reasons underlie the present study. First, existing research rarely examines how family doctor contracting influences patients' graded utilization of different healthcare institution levels. Prior studies have largely focused on primary care usage, awareness of the family doctor system, signing determinants, chronic disease management outcomes, and cost control effects, without exploring hierarchical healthcare patterns. Second, methodological limitations prevail: most studies rely on cross-sectional data, small sample sizes, short study durations, and lack control groups, limiting causal inferences. Third, previous research has often employed simple comparisons and descriptive analyses without addressing endogeneity or confounding, which are critical for establishing causality.

Thus, the objective of this study was to investigate the impact of family doctor contracting on the graded utilization of healthcare services among diabetic patients, using real-world longitudinal data.

## Methods

### Study design

This was a retrospective cohort study. Diabetic patients were identified based on the International Classification of Diseases, 10th Revision (ICD-10) codes. Participants were categorized into contracted and non-contracted groups according to whether they had signed a family doctor contract.

Diabetic patients with and without family doctor contracts often differ in baseline characteristics (e.g., age, income, health status). Direct outcome comparison between these groups risks selection bias, complicating isolation of the family doctor system's net effect on healthcare utilization. After carefully evaluating the core methodological rationale and data specifications, the inverse probability-weighted regression adjustment (IPWRA) approach was selected as the most appropriate analytical method. The IPWRA method, widely used for causal inference, addresses baseline imbalances through inverse probability weighting, approximating a randomized trial population. By eliminating selection bias and controlling for confounding variables, IPWRA enables estimation of the intervention's net causal impact. Given this observational study's inherent baseline disparities between contracted and non-contracted patients, IPWRA was selected to balance group characteristics and quantify the system's true causal effect (17–19).

### Data sources and population selection

Data were extracted from the Health Information Center Database of S City, which consolidates municipal-level patient data, including demographics, contracting status, lifestyle behaviors, chronic disease records, healthcare utilization across primary, secondary, and tertiary hospitals, healthcare costs, mortality, and complication information.

**Figure 1:**
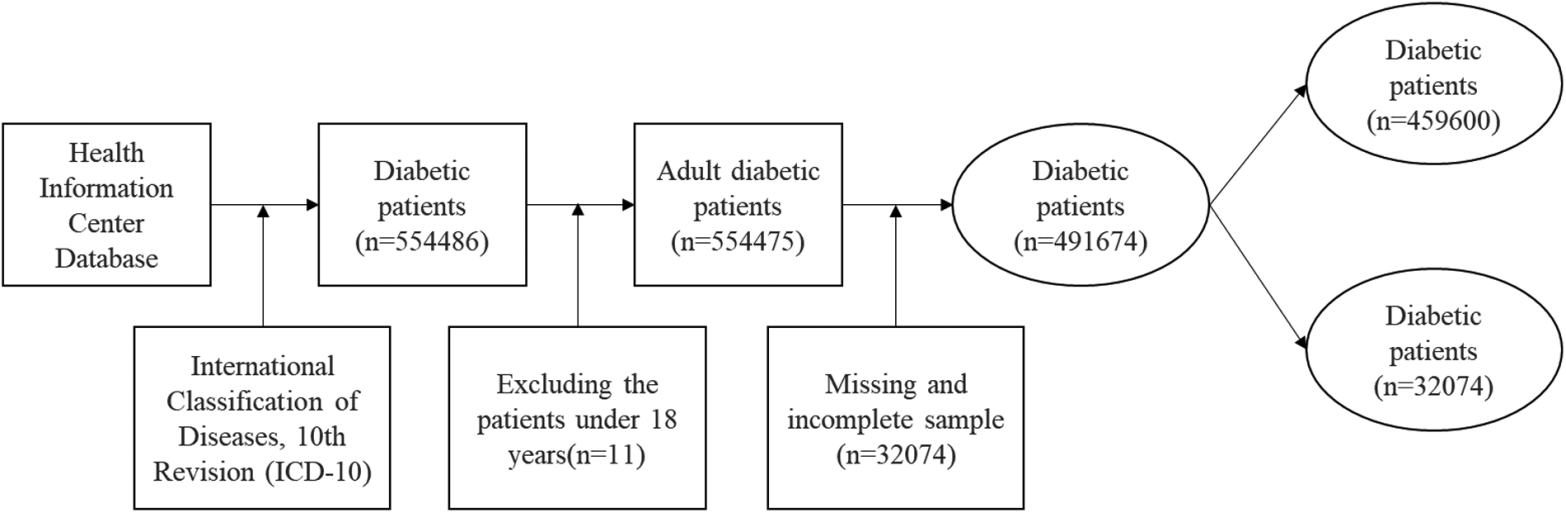
Flow chart of data sources and sample selection.

We retrieved annual patient records from January 1, 2014, to December 31, 2021, yielding up to eight observations per patient. A total of 554,486 diabetic patients were identified. After excluding patients under 18 years and cases with missing data (*n* = 62,801), the final sample included 491,674 patients: 459,600 contracted and 32,074 non-contracted. Inclusion criteria include: (1) Patients diagnosed with diabetes according to ICD-10; (2) Patients aged 18 years or older with diabetes; (3) Complete baseline data; (4) Complete health service utilization data (number of outpatient and inpatient visits). Exclusion criteria include: (1) Baseline data with >20% missing values for key variables (e.g., BMI, smoking status, blood pressure values, etc.); (2) Patients aged under 18 years with diabetes; (3) Duplicate data exported from the system; (4) Patients with incomplete healthcare utilization data; (5) Patients who were lost to follow-up during the follow-up period. This study restricted the research sample according to the following criteria (Figure 1):

### Research variables

#### Dependent variables

Eight outcome measures, including total outpatient visits, total inpatient visits, and outpatient/inpatient visits by hospital level (primary, secondary, tertiary).

#### Independent variables

The main independent variable was contracting status (1 = contracted, 0 = non-contracted). Control variables included gender (male and female), age group (<65and ≥85), residential location (urban and outskirts), ethnicity (Han vs. others), BMI categories (<<18.5, 18.5–23.9, 24–27.9, ≥28), smoking status (yes or no), alcohol consumption (yes or no), blood pressure levels (normal, upper normal range, grade 1, grade 2, grade 3), and physical activity status (yes or no).

### Statistical analyses

IPWRA and Zero-Inflated Negative Binomial Regression (ZINB) were used in this study, the two methods were combined to estimate the net effect. IPWRA used to minimize the baseline bias to increase the reliability of differences in comparisons between the two groups, according to gender, age, BMI, blood, smoking, frequency of alcohol, regular activity and area as matching variables. Generate to propensity scores, for individuals who have signed up for a family doctor, the weight w = 1/p, for those who have not signed up for a family doctor, the weight w = 1/(1-p). Given that the utilization of health services is count data and the sample mean is not equal to the variance in this study, a negative binomial regression model was selected (20, 21). According to the Vuong test, if| Vuong |≥1.96, then a zero-inflated negative binomial regression model was chosen. For all data sets, differences were considered statistically significant when *p* value < 0.05. All analyses were performed using STATA software (version 16.0).

## Results

### Baseline characteristics

As shown in Table 1, contracted patients were older (mean 66.31 years), predominantly female (52.08%), and had higher BMI and blood pressure levels compared to non-contracted patients. Non-contracted patients tended to be younger and more likely to reside in urban areas, highlighting baseline imbalances that justified the use of IPWRA.

**Table 1:** Descriptive statistics for study participants.

Variable	Total (*N* = 491,674)	Contracted (*N* = 459,600)	Non-contracted (*N* = 32,074)	*P*
Gender (%)				0.000^a^
Male	48.07	47.92	50.29	
Female	51.93	52.08	49.71	
Age (M ± SD, year)	66.30 ± 10.15	66.31 ± 10.01	66.24 ± 11.95	0.000^b^
Age classification (%)				0.000^a^
<65	42.76	33.31	50.52	
≥65	57.24	66.69	49.48	
Region(%)				0.000^a^
Urban	50.54	49.46	63.27	
Suburban	49.73	50.54	36.73	
Ethnicity (%)				0.000^a^
Han	96.41	96.21	99.21	
Other	3.59	3.79	0.79	
Height (M ± SD, cm)	163.61 ± 7.84	163.56 ± 7.83	164.27 ± 7.83	0.476^b^
Height classification (%)				0.000^a^
<160	30.78	30.99	27.85	
160–169	40.64	40.66	40.38	
≥170	28.58	28.35	31.77	
Weight（M ± SD, kg）	65.35 ± 9.67	65.34 ± 9.66	65.38 ± 9.78	0.000^b^
Weight classification (%)				0.000^a^
<55	10.73	10.72	10.87	
55–64	35.27	35.29	34.91	
65–74	36.46	36.47	36.30	
≥75	17.54	17.52	17.91	
BMI(M ± SD, kg/m^2^)	24.37 ± 2.92	24.38 ± 2.91	24.18 ± 2.93	0.004^b^
BMI classification (%)				0.000^a^
<18.5	1.24	1.20	1.28	
18.5–23.9	46.44	46.17	46.67	
24.0–27.9	41.91	42.22	41.65	
>28.0	10.41	10.41	10.41	
SBP (M ± SD, mmHg)	128.89 ± 7.82	128.90 ± 7.86	128.68 ± 7.30	0.000^b^
SBP classification (%)				0.000^a^
<120	4.99	5.06	4.92	
120–139	89.79	89.65	89.91	
140–159	4.50	4.57	4.44	
160–179	0.61	0.60	0.62	
≥180	0.11	0.11	0.12	
Drinking (%)				0.000^a^
Yes	9.97	10.04	8.98	
No	90.03	89.96	91.02	
Smoking (%)				0.000^a^
Yes	10.05	10.13	8.92	
No	89.95	89.87	91.08	
Regular Activity (%)				0.000^a^
Yes	29.29	29.58	25.23	
No	70.71	70.42	74.77	

^a^ Pearson chi-square test.

^b^ student-*t* test.

### Health service utilization

Table 2 summarizes healthcare utilization patterns. The average number of outpatient visits among diabetic patients was 32.61 visits per year, while the average inpatient hospitalization rate was 0.38 admissions per year. Contracted patients had slightly fewer outpatient visits but higher inpatient admissions compared to non-contracted patients. Patients in the contracted group had more visits to community health centers (20.92 vs. 19.95) but fewer visits to secondary and tertiary hospitals.

**Table 2:** Health service utilization in patients with diabetes (M ± SD).

Variable	Total		Contracted		Non-contracted		*P*
	Outpatient	Inpatient	Outpatient	Inpatient	Outpatient	Inpatient	
Total visits	32.61 ± 32.31	0.38 ± 1.28	32.47 ± 31.60	0.42 ± 1.33	34.63 ± 41.04	0.35 ± 1.24	0.000^a^
Community health centers visits	20.85 ± 24.25	0.05 ± 0.58	20.92 ± 23.78	0.06 ± 0.65	19.95 ± 30.28	0.04 ± 0.52	0.000^a^
Secondary hospitals visits	6.75 ± 12.50	0.17 ± 0.82	6.65 ± 12.20	0.19 ± 0.84	8.22 ± 16.13	0.16 ± 0.80	0.000^a^
Tertiary hospitals visits	5.00 ± 10.91	0.16 ± 0.72	4.91 ± 10.61	0.15 ± 0.71	6.44 ± 14.45	0.15 ± 0.72	0.000^a^

^a^ Pearson chi-square test.

### Regression results

In outpatient care, IPWRA analysis revealed that the family doctor system exerted a statistically significant negative effect on health service utilization across all care levels. Compared to non-contracted patients, contracted diabetics showed a 7.30% reduction in total outpatient visits (IRR = 0.92, *P* < 0.01), corresponding to 2.43 fewer outpatient visits. The reduction was most pronounced in secondary hospitals (−19.53%, IRR = 0.82, *P* < 0.01) and tertiary hospitals (−17.02%, IRR = 0.84, *P* < 0.01), followed by primary care facilities (−4.90%, IRR = 0.95, *P* < 0.01), demonstrating greater utilization decline in higher-level hospitals, as shown in Table 3 and Supplementary S1.

**Table 3:** The results for the impact of the family doctor system on outpatient visits in patients with diabetes.

Variables	Total		Community health centers outpatient visits		Secondary hospitals outpatient visits		Tertiary hospitals outpatient visits	
	IRR	95%CI	IRR	95%CI	IRR	95%CI	IRR	95%CI
Contract Status								
Non-contracted	Ref.							
Contracted	0.92***	0.92–0.93	0.95***	0.95–0.96	0.82***	0.81–0.82	0.84***	0.84–0.85
Gender								
Femal	Ref.							
Male	0.81***	0.80–0.81	0.79***	0.79–0.80	0.82***	0.82–0.83	0.89***	0.89–0.90
Age								
<65	Ref.							
≥65	1.52***	1.52–1.53	1.65***	1.65–1.66	1.30***	1.30–1.31	1.20***	1.20–1.21
Region								
Urban	Ref.							
Suburban	0.76***	0.76–0.77	0.80***	0.80–0.81	0.86***	0.86–0.87	0.44***	0.43–0.44
BMI								
<18.5	Ref.							
18.5–23.9	0.97***	0.96–0.98	1.01***	1.00–1.02	0.94***	0.93–0.96	0.89***	0.87–0.91
24.0–27.9	0.99	0.98–1.00	1.04***	1.03–1.05	0.96***	0.95–0.98	0.87***	0.55–0.89
>28.0	1.04***	1.03–1.05	1.09***	1.07–1.10	1.04***	1.02–1.06	0.88***	0.86–0.90
SBP								
<120	Ref.							
120–139	1.00***	1.00–1.01	1.01***	1.01–1.02	0.97***	0.96–0.97	1.02***	1.01–1.03
140–159	1.01***	1.01–1.02	1.05***	1.04–1.06	0.96***	0.94–0.97	0.90***	0.89–0.91
160–179	1.01***	1.01–1.02	1.09***	1.07–1.10	0.87***	0.85–0.89	0.81***	0.78–0.83
≥180	0.98	0.96–1.01	1.04***	1.01–1.07	0.90***	0.85–0.95	0.76***	0.71–0.82
Drinking								
Yes	Ref.							
No	0.94***	0.94–0.95	0.98***	0.97–0.98	0.91***	0.90–0.92	0.85**	0.84–0.86
Smoking								
Yes	Ref.							
No	0.91***	0.91–0.92	0.93***	0.93–0.94	0.92***	0.91–0.93	0.84***	0.84–0.86
Regular Activity								
Yes	Ref.							
No	1.08***	1.07–1.08	1.10***	1.10–1.11	1.05***	1.05–1.06	1.01***	1.00–1.02

*P < 0.05; **P < 0.01; ***P < 0.001.

IRR, incidence rate ratio; CI, confidence intervals; BMI, body mass index; SBP, systolic blood pressure.

For inpatient care, a contrasting pattern emerged. Contracted patients exhibited an 8.2% increase in total hospitalizations (IRR = 1.08, *P* < 0.01), equivalent to 0.03 additional hospitalization days. The rise was predominantly observed in primary hospitals (+26.88%, IRR = 1.30, *P* < 0.01), with secondary hospitals showing moderate growth (+8.3%, IRR = 1.08, *P* < 0.01) and tertiary hospitals minimal change (+2.30%, IRR = 1.02, *P* < 0.01). The hospitalization increase was most significant at the primary care level, as shown in Table 4 and Supplementary S2.

**Table 4:** The results for the impact of the family doctor system on inpatient visits in patients with diabetes.

Variables	Total		Community health centers inpatient visits		Secondary hospitals inpatient visits		Tertiary hospitals inpatient visits	
	IRR	95%CI	IRR	95%CI	IRR	95%CI	IRR	95%CI
Contract Status								
Non-contracted	Ref.							
Contracted	1.08***	1.07–1.09	1.30***	1.27–1.34	1.08***	1.07–1.09	1.02***	1.01–1.03
Gender								
Femal	Ref.							
Male	1.05***	1.04–1.06	0.95***	0.93–0.98	1.03***	1.02–1.04	1.14***	1.13–1.15
Age								
<65	Ref.							
≥65	1.52***	1.51–1.53	3.12***	3.03–3.20	1.54***	1.52–1.55	1.22***	1.21–1.23
Region								
Urban	Ref.							
Suburban	0.91***	0.91–0.92	1.19***	1.16–1.22	1.20***	1.19–1.21	0.61***	0.60–0.61
BMI								
<18.5	Ref.							
18.5–23.9	0.82***	0.80–0.85	0.61***	0.55–0.68	0.85***	0.82–0.89	0.88***	0.84–0.92
24.0–27.9	0.82***	0.79–0.84	0.57***	0.52–0.64	0.87***	0.84–0.91	0.87***	0.83–0.91
>28.0	0.89***	0.86–0.92	0.61***	0.55–0.68	0.99	0.95–1.03	0.90***	0.86–0.9
SBP								
<120	Ref.							
120–139	0.97***	0.96–0.99	0.82***	0.78–0.87	0.99	0.97–1.01	1.01	0.98–1.03
140–159	0.93***	0.91–0.95	0.73***	0.67–0.78	0.99	0.96–1.02	0.92***	0.89–0.95
160–179	0.89***	0.85–0.93	0.57***	0.49–0.66	0.94	0.88–0.99	0.93	0.87–0.99
≥180	0.91***	0.84–0.99	0.74***	0.56–0.97	1.03***	0.92–1.16	0.83***	0.73–0.95
Drinking								
Yes	Ref.							
No	0.81***	0.80–0.82	0.66***	0.63–0.70	0.82***	0.80–0.83	0.83**	0.81–0.85
Smoking								
Yes	Ref.							
No	0.96***	0.95–0.98	0.93***	0.88–0.98	1.01	0.99–1.03	0.93***	0.81–0.85
Regular Activity								
Yes	Ref.							
No	1.24***	1.23–1.25	1.65***	1.60–1.69	1.28***	1.27–1.29	1.10***	1.09–1.12

* *P* < 0.05; **P < 0.01; ***P < 0.001.

IRR, incidence rate ratio; CI, confidence Intervals; BMI, body mass index; SBP, systolic blood pressure.

ZINB models confirmed the robustness of these findings, as shown in Supplementary Tables S3 and S4.

The difference between the standardized differences of the original data before inverse probability weighting and the two groups of data after inverse probability weighting tends to be close to 0, and the variance ratio tends to be close to 1, which indicates that the baseline difference between the samples of the contracted group and the uncontracted group has been balanced very well, which effectively reduces the selection bias and the effect of the confounding caused by the non-randomness of contracting behaviours, and enhances the comparability of the samples of the contracted group and the uncontracted group, as shown in Table 5.

**Table 5:** Results of the balance test of the distribution of covariates before and after inverse probability weighting.

Variable	Standardized differences		Variance ratio	
	Before matching	After matching	Before matching	After matching
Gender	0.028	0.000	0.997	0.999
Age				
≥65	0.352	0.000	0.888	1.000
BMI				
18.5–23.9	0.009	0.000	0.998	1.000
24.0–27.9	0.111	0.000	1.003	0.999
>28.0	0.000	0.000	1.000	1.000
SBP				
120–139	0.008	0.000	1.021	0.999
140–159	0.006	0.000	1.027	0.999
160–179	0.002	0.000	0.971	0.998
≥180	0.002	0.000	0.915	0.997
Drinking Status (%)				
Yes	0.010	0.000	0.973	0.998
Smoking Status (%)	0.009	0.000	0.970	0.997
Yes	0.020	0.000	0.947	0.998
Regular Activity	0.012	0.000	0.988	0.999
Region	0.142	0.000	0.999	1.000

## Discussion

To the best of our knowledge, this is among the few studies assessing the long-term net effect of the family doctor system on healthcare utilization among diabetic patients in China using real-world longitudinal data. The findings suggest that the family doctor system was associated with a significant decrease in outpatient visits for diabetic patients, decreased by 3.7% in community health centers, 19.2% in secondary hospitals and 16.6% in tertiary hospitals. Regarding inpatient visits, the research appears that compare with non-contracted group, higher utilization of inpatient services among diabetic patients in the contracted group, and increase in inpatient services mainly concentrated in community health centers. The family doctor system in China aims to provide basic medical care, public health, health management, convenient drug refills, optimization of referral services for chronic patients, formation of an orderly order of medical care and a mechanism for the gradual utilization of resources, address their basic health service needs, and promote a hierarchical system of diagnosis and treatment. These results are consistent with previous studies reporting reduced outpatient utilization following implementation of family doctor services (22, 23), but contrast with others suggesting increased outpatient visits (24, 25).

Here are the reasons for the differences and similarities between the study findings and those of previous studies. First of all, the impact of long-term prescription policy of family doctor system on outpatient visits (26). S City is a coastal city in eastern China where the government is actively promoting the high-quality development of contracted family doctor services. To strengthen the role of primary care as the first point of contact and to meet the long-term medication needs of patients, S City gives priority to eligible contracted patients with chronic illnesses by providing long-term prescription services and prescribing prolonged dosages of medication, which reduces the frequency of patients’ visits to the doctor's office.

Secondly, with a developed economic and informatization base, S City has developed the “Internet+” family doctor contracting service information platform and the Health Cloud APP, which allows patients to ask for consultation and advice online and prescribe medication online from their smartphones, thus reducing the number of outpatient visits by eliminating the need for patients to go to the hospital for consultation (6).

Thirdly, the decrease in the number of outpatient visits and the increase in the number of hospitalizations of contracted diabetic patients may be due to the transfer of outpatient services to inpatient services, and there is published literature confirming higher rates of hospitalization of patients in European countries with well-developed healthcare delivery systems and in those countries/regions where patients are more likely to have access to care (26).

Fourthly, as diabetics are at a higher risk of having complications (diabetic foot disease, diabetic nephropathy, diabetic retinopathy, diabetic neuropathy, etc.), signing up for a family doctor who can detect complications at an early stage of the disease will increase the number of hospital admissions.

Fifth, the outpatient reimbursement rate is lower than the inpatient reimbursement rate in S city. This may lead to an increase in the number of hospitalizations for contracted patients.

Sixth, the annual hospitalization rate of Chinese residents in 2023 was 21.4%, and the annual hospitalization rate of residents in S City was also high, which may have induced demand for overhospitalization and increased the frequency of hospitalization of diabetic patients.

Adjusting the family doctor system to reduce hospitalization rates while maintaining efficiency necessitates a multifaceted strategy integrating prevention, management, collaboration, and technological innovation. Key measures include: strengthening early complication screening for diabetic patients, developing personalized health plans to control disease onset/progression, and reducing diabetes-related hospitalizations; establishing a health-outcome-oriented digital diabetes management platform with performance evaluation linked to health indicators; leveraging digital systems to visualize management effectiveness, enabling real-time outcome monitoring by clinicians, administrators, and policymakers; implementing clinical decision support and remote monitoring systems to facilitate timely interventions; developing clear referral protocols and optimizing Shanghai's “decentralized appointment allocation” policy to establish bidirectional referral pathways between primary and specialist care; and providing regular training to enhance family doctors' capacity in managing common chronic diseases, thereby reducing avoidable hospital transfers (13, 27).

### Limitations

First, although we controlled for major confounders, we were unable to include certain socioeconomic variables such as education level, income level, treatment compliance, referral patterns and resource accessibility, which may correlate with both family doctor contracting behavior and health service utilization. Second, the mechanisms underlying the observed decrease in outpatient visits and increase in hospitalizations among contracted diabetic patients were not directly explored. While we discussed possible explanations, future research using methods such as structural equation modeling is needed to better elucidate these mechanisms. Third, as the study was conducted in a pilot city with higher socioeconomic status and an earlier adoption of the family doctor system than most parts of China, the findings, while applicable to areas with similarly advanced systems, may not be generalizable to rural or less-developed regions where registration rates are lower. Four, due to data limitations, we have not yet obtained information on changes in the contract status of diabetic patients, the analysis assumes static grouping, which could introduce misclassification bias. Static grouping may underestimate or overestimate the effect, we consider this limitation to be an area for improvement in future studies. Five, Shanghai is one of China's most economically developed regions. Factors such as the concentration of medical resources, developed public transportation network, advanced medical equipment, and high levels of resident health literacy may have influenced the study results and limit their applicability to rural or less-developed regions. Future research will conduct stratified comparative studies between developed and underdeveloped regions.

### Strengths

Despite its limitations, this study provides valuable insights. Several strengths should be recognized. First, regarding study design, we tracked the impact of family doctor contracting on health service utilization over an extended period, using eight years of panel data. This approach avoids the limitations associated with cross-sectional designs and shorter follow-up periods. Second, in terms of study population selection, this study employed a census of diabetic patients in S City, thereby overcoming the patient selection bias commonly associated with sampling methods. Finally, from a methodological perspective, the decision to contract with a family doctor is not random but is closely related to patients' health status, personal preferences, and other factors, introducing the risk of endogenous selection bias and potential baseline differences between contracted and non-contracted patients. To address this, we applied the Inverse Probability Weighted Regression Adjustment (IPWRA) method to mitigate selection bias and further evaluated the impact of family doctor contracting using zero-inflated negative binomial regression analysis. These methodological strengths likely enhanced the robustness and accuracy of the study findings.

## Conclusion

This study evaluated the net effect of the family doctor system on hierarchical healthcare utilization among diabetic patients. Family doctor contracting was associated with significantly reduced outpatient visits across all hospital levels but increased inpatient admissions, particularly at primary care institutions. Future research should further explore underlying mechanisms and assess impacts on patient outcomes and healthcare costs to support evidence-based policymaking.

## Data Availability

The data analyzed in this study was obtained from Johns Hopkins University, the following licenses apply by Johns Hopkins University. Requests to access these datasets should be directed to Yin Fan, gpfanyin@jhu.edu.
